# LIVING ALONE WITH DEMENTIA: FINDINGS FROM THE IDEAL COHORT

**DOI:** 10.1093/geroni/igz038.155

**Published:** 2019-11-08

**Authors:** Linda Clare, Linda Clare, Anthony Martyr, Catherine Quinn, Christina Victor, Fiona Matthews

**Affiliations:** 1 University of Exeter, Exeter, England, United Kingdom; 2 Bradford University, Bradford, England, United Kingdom; 3 Brunel University London, Uxbridge, England, United Kingdom; 4 NewcCastle University, Newcastle, England, United Kingdom

## Abstract

We aimed to better understand the profile of people living alone with mild-to-moderate dementia in the UK and to identify any systematic differences between those living alone and those living with others. We analysed cross-sectional data from 1541 people with mild-to-moderate dementia participating in the IDEAL cohort at the first wave of assessment. There were 285 participants (18.5%) living alone and 1256 (81.5%) living with others, usually a spouse/partner. Among those living alone, 145 (50.9%) had no care partner participating in the study, and 56 (19%) had received no help from a relative or friend in the past week. People living alone were older on average than those living with others, reported fewer functional difficulties, had slightly smaller social networks, engaged in fewer cultural activities, and experienced slightly more loneliness. People living alone had lower satisfaction with life scores, but quality of life scores did not differ between the groups.

